# Outcomes of Patients With Early and Locally Advanced Lung Cancer: Protocol for the Italian Lung Cancer Observational Study (LUCENT)

**DOI:** 10.2196/57183

**Published:** 2024-10-08

**Authors:** Luca Bertolaccini, Oriana Ciani, Marco Lucchi, Francesco Zaraca, Alessandro Bertani, Roberto Crisci, Lorenzo Spaggiari, LUCENT Study Group

**Affiliations:** 1 Department of Thoracic Surgery IEO, European Institute of Oncology IRCCS Milan Italy; 2 Centre for Research on Health and Social Care Management (CERGAS) Bocconi School of Management Bocconi University Milan Italy; 3 Division of Thoracic Surgery University of Pisa Pisa Italy; 4 Division of Thoracic Surgery Regional Hospital Bozen/Bolzano Italy; 5 Unit of Thoracic Surgery and Lung Transplantation Department for the Treatment and Study of Cardiothoracic Diseases and Cardiothoracic Transplantation Istituto Mediterraneo per i Trapianti e Terapie ad Alta Specializzazione (IRCCS-ISMETT) Palermo Italy; 6 Division of Thoracic Surgery University of L'Aquila L'Aquila Italy; 7 Department of Oncology and Hemato-Oncology University of Milan Milan Italy; 8 see Authors' Contributions

**Keywords:** lung cancer, quality of life, observational study, economic aspects, multicenter study

## Abstract

**Background:**

Lung cancer, predominantly non-small cell lung cancer (NSCLC), remains a formidable challenge, necessitating an in-depth understanding of evolving treatment paradigms. The Italian Lung Cancer Observational Study (LUCENT) addresses this need by investigating the outcomes of patients with early and locally advanced lung cancer in Italy.

**Objective:**

With a focus on real-world data and patient registries, this study aims to provide comprehensive insights into clinical, psychosocial, and economic impacts, contributing to informed decision-making in health care.

**Methods:**

LUCENT is a prospective observational multicenter cohort study enrolling patients eligible for minimally invasive manual, robot-assisted, or traditional open surgery. The study will develop a web-based registry to collect longitudinal surgical, oncological, and socioeconomic outcome data. The primary objectives include performance assessment through the establishment of national benchmarks based on risk-adjusted outcomes and processes of care indicators. The secondary objectives encompass economic and psychosocial impact assessments of innovative technologies and treatment pathways. The multicenter design ensures a diverse and representative study population.

**Results:**

The evolving landscape of NSCLC treatment necessitates a nuanced approach with consideration of the dynamic shifts in therapeutic strategies. LUCENT strives to fill existing knowledge gaps by providing a platform for collecting and analyzing real-world data, emphasizing the importance of patient-reported outcomes in enhancing the understanding of the disease. By developing a web-based registry, the study not only facilitates efficient data collection but also addresses the limitations of traditional methods, such as suboptimal response rates and costs associated with paper-and-pencil questionnaires. Recruitment will be conducted from January 01, 2024, to December 31, 2026. Follow-up will be performed for a minimum of 2 years. The study will be completed in the year 2028.

**Conclusions:**

LUCENT’s potential implications are substantial. Establishing national benchmarks will enable a thorough evaluation of outcomes and care processes, guiding clinicians and policymakers in optimizing patient management. Furthermore, the study’s secondary objectives, focusing on economic and psychosocial impacts, align with the contemporary emphasis on holistic cancer care. Insights gained from this study may influence treatment strategies, resource utilization, and patient well-being, thereby contributing to the ongoing refinement of lung cancer management.

**Trial Registration:**

ClinicalTrials.gov NCT05851755; https://clinicaltrials.gov/study/NCT05851755. ISRCTN 67197140; https://www.isrctn.com/ISRCTN67197140

**International Registered Report Identifier (IRRID):**

PRR1-10.2196/57183

## Introduction

### Background and Rationale

Most lung cancer cases involve non-small cell lung cancer (NSCLC), while small cell cancer represents the minority of cases. To date, limited percentages of NSCLC cases are diagnosed in the early stage (in which patients are potential candidates for surgery, possibly followed by chemotherapy, to reduce the risk of recurrence) or in the locally advanced stage (in which the treatment is based on the use of chemotherapy, radiotherapy, and possibly, upon their completion, immunotherapy). The therapeutic strategy in patients with advanced NSCLC has changed in recent years. Until about 10 years ago, chemotherapy was the only option available; however, it was characterized by limited effectiveness. In recent years, the 2 crucial therapeutic “revolutions” we have witnessed in medical oncology (molecularly targeted drugs and then immunotherapy) have played essential roles in treating these patients. Some molecularly targeted drugs (primarily epidermal growth factor receptor [EGFR] inhibitors and then drugs directed against other molecular alterations) are superior to chemotherapy as a first-choice treatment, but the use of these drugs is limited to cases in which the tumor has those specific molecular alterations. Molecular analyses aimed at identifying these alterations in the tumor tissue represent a fundamental part of the diagnosis, which precedes the best treatment choice for each patient. In the next few years, we will probably see the continuation of those therapeutic “revolutions” mentioned above with the availability of new molecularly targeted drugs. These approaches will increase the therapeutic possibilities to be used in sequence after the failure of the approaches already available today [1]. Still, in some cases, they will allow a “targeted” treatment in the presence of molecular alterations for which no target drugs are available in clinical practice until now. Immunotherapy first established itself (about 5 years ago) as an effective treatment in patients who had already failed chemotherapy [1]. Subsequently, it was proven to be superior to chemotherapy as the first-choice treatment in cases characterized by high expression of the PD-L1 marker. Finally, significant results have been obtained in recent years by combining chemotherapy and immunotherapy, even in patients with low or absent PD-L1 expression. It is conceivable that in the coming years, based on a series of clinical trials recently conducted or still in progress, innovative drugs (targeted therapies and immunotherapy) will have essential roles in the treatment of early stages of cancer (used before surgery or after surgery) to reduce the risk of disease recurrence and hopefully increase the chances of recovery. The 5-year survival rate of lung cancer is low (16% in men and 23% in women), and it is at the bottom of the survival ranking, reminding us that, despite the critical progress made in recent years, there is a long way ahead regarding its treatment [2-4].

Globally, there is an increasing trend to use real-world data to inform decision-making in health care, and patient registries are regarded as a typical example of real-world data. A patient registry can be defined as “an organized system that uses observational study methods to collect uniform data (clinical and other) to evaluate specified outcomes for a population defined by a particular disease, condition, or exposure, and that serves one or more predetermined scientific, clinical, or policy purposes” [5,6]. While regulatory agencies can use real-world data collection for postmarketing surveillance and risk assessment, payers and reimbursement agencies are consistently considering real-world evidence to make or revise their recommendations. To this end, the information collected in patient registries can extend from appropriate treatment strategies to effectiveness and cost-effectiveness assessments in real-world clinical practice [7,8].

Furthermore, achieving and maintaining optimal well-being and health-related quality of life (HRQoL) have become essential objectives of cancer treatment, rehabilitation, and aftercare across the cancer continuum. The availability of patient-reported outcomes (PROs) is critical in achieving these goals. In the past, patient registries have provided clinicians and researchers with a wealth of clinical data (eg, stage and primary treatment) on cancer patients. However, data on PROs are not yet routinely available. PROs have been collected using paper-and-pencil questionnaires, with suboptimal response rates, high costs, and overall process efficiency. Online administration of questionnaires has several advantages compared with paper-and-pencil questionnaires, including convenience for the participant, potentially significant cost savings, data collection efficiency, and high data quality [9-12].

Therefore, we propose to develop a web-based registry to collect longitudinal data on the clinical, psychosocial, and economic impacts of lung cancer treatment in Italy.

### Objectives

#### Primary Objectives

The project involves a web-based registry developed to facilitate data collection and evaluation of several different surgical, oncological, and socioeconomic outcomes of lung cancer patients diagnosed in early and locally advanced stages in Italy.

The primary objective is to generate data relevant to performance assessment through the development of national performance benchmarks based on the analysis of risk-adjusted outcomes and the processes of care indicators (eg, How does individual center X compare to the national standard in terms of length of stay for a certain subgroup of patients?).

#### Secondary Objectives

The registry can be used simultaneously for multiple studies with different purposes. Among the secondary objectives are the following: (1) Economic impact assessment of innovative technologies or treatment pathways (eg, Do different surgical medical devices influence health care resource utilization for a single episode of hospitalization?) and (2) Psychosocial impact assessment of innovative technologies or treatment pathways, for example, minimally invasive surgery (eg, Do new targeted treatments have a beneficial or adverse impact on PROs?).

### Trial Design

The Italian Lung Cancer Observational Study (LUCENT) is a prospective observational multicenter cohort study enrolling lung cancer patients who are candidates for minimally invasive manual, robot-assisted, or traditional open surgery. This study aims to investigate the outcomes of patients with early and locally advanced lung cancer in Italy. By using real-world data and patient registries, the study seeks to provide comprehensive insights into the clinical, psychosocial, and economic impacts of lung cancer treatment. The study’s observational nature allows for data collection in a naturalistic setting without intervention or manipulation by the researchers. The list of collaborating institutions has been provided in Multimedia Appendix 1.

## Methods

### Study Setting

This multicenter study will include community clinics and academic centers located in Italy. This protocol has been presented in compliance with the SPIRIT (Standard Protocol Items: Recommendations for Interventional Trials) checklist [13].

### Eligibility Criteria

#### Inclusion Criteria

The inclusion criteria are as follows:

- Diagnosis of lung cancer: Patients must meet the following criteria for the diagnosis of lung cancer: (1) confirmation of lung cancer through histopathological examination or imaging studies and (2) specific ICD-10 codes for lung cancer that include but are not limited to C34.0 (main bronchus), C34.1 (upper lobe, lung), C34.2 (middle lobe, lung), C34.3 (lower lobe, lung), C34.8 (overlapping lesion of the lung), and C34.9 (lung, unspecified).
- Age criteria: Patients must be aged 18 years or older at the time of enrollment.
- Informed consent: Patients must provide written informed consent to participate in the study.
- Communication requirement: Patients should provide an email address at the time of enrollment for follow-up communication and the exchange of questionnaires.
- Language proficiency: Patients should be able to understand and communicate in Italian.

#### Exclusion Criteria

The exclusion criteria are as follows:

- Language proficiency: Patients are unable to understand or communicate in Italian.

### Who Will Take Informed Consent?

Qualified health care professionals will conduct the informed consent process at each participating institution in this study. These professionals will ensure that potential participants are fully informed about the study’s purpose, procedures, and possible risks and benefits, and their rights as participants. They will also address any questions or concerns raised by the participants and obtain written informed consent from those who voluntarily choose to participate in the study. The informed consent process will adhere to ethical guidelines and regulatory requirements to safeguard the rights and well-being of the participants.

### Additional Consent Provisions for the Collection and Use of Participant Data and Biological Specimens

We will conduct the trial according to the ICH Good Clinical Practice (GCP) guidelines. Accurate and consistent records are essential to a cooperative study.

The clinical and surgical data collected in a standardized endorsed data set can be downloaded locally and used for internal quality analyses or institutional research. The data collection methodology will use a website platform that meets international data privacy protection standards and rules. Notably, data are anonymously reported, independently accessed, and encrypted for other users.

For joining the database, the login of each institution will be provided after downloading and completing an application form from the project homepage and directly sending an email to the study coordinator. Investigators will access the patient medical records and enter the required data into the database. Protected health information will not be reused or disclosed to any other person or entity, except as required by law, for authorized research project oversight.

In compliance with the ICH-GCP guidelines, the investigator or institution will maintain all electronic case report forms, all source documents that support the data collected from each subject, all study documents as specified in ICH-GCP Section 8 (Essential Documents for the Conduct of a Clinical Trial), and all study documents as determined by the applicable regulatory requirements. The investigator or institution will take measures to prevent accidental or premature destruction of these documents. Essential documents must be retained until at least 25 years after the last approval of a marketing application in an ICH region and until there are no pending or contemplated marketing applications in an ICH region or until at least 15 years have elapsed since the formal discontinuation of clinical development of the investigational product. These documents will be retained longer if required by the applicable regulatory requirements or an agreement with the sponsor. The sponsor is responsible for informing the investigator or institution when these documents no longer need to be retained.

If the responsible investigator retires, relocates, or withdraws from the responsibility of keeping the study records for other reasons, custody must be transferred to a person who will accept the duty. The sponsor must be notified in writing of the name and address of the new custodian. Under no circumstance shall the investigator relocate or dispose of any study documents before obtaining written approval from the sponsor. If it becomes necessary for the sponsor or the appropriate regulatory authority to review any documentation relating to this study, the investigator must permit access to such reports.

### Interventions

#### Explanation for the Choice of Comparators

The choice of comparators in this study is driven by the need to assess and compare various surgical interventions for lung cancer patients. The study aims to enroll patients eligible for minimally invasive manual, robot-assisted, or traditional open surgery, reflecting the diverse landscape of surgical approaches in clinical practice.

The rationale for including these comparators is rooted in the evolving nature of lung cancer treatment, where different surgical modalities are employed based on factors such as tumor characteristics, patient condition, and advancements in surgical techniques. Minimally invasive surgeries, including robot-assisted procedures, have gained prominence for their potential benefits, such as reduced postoperative pain, shorter hospital stay, and quicker recovery compared to traditional open surgery.

By including these comparators, this study seeks to evaluate and compare the outcomes of these diverse surgical interventions. This comprehensive approach allows for a nuanced understanding of the effectiveness and potential differences in results associated with each surgical modality. It also enables the study to contribute valuable insights into the evolving landscape of lung cancer treatment, guiding future clinical decisions and optimizing patient care.

The choice of comparators aligns with the study’s overarching objective to generate data for performance assessment. This provides a basis for national benchmarks and facilitates a thorough analysis of outcomes and care processes. This approach ensures that the study captures the diversity of surgical practices in the real-world setting, reflecting the dynamic nature of lung cancer treatment and contributing to the broader knowledge base in the field.

#### Intervention Description

The interventions in this study encompass a spectrum of surgical approaches for lung cancer patients diagnosed in the early and locally advanced stages.

Regarding the rationale for intervention selection, the choice of interventions reflects the diversity of surgical practices in treating lung cancer. Each intervention has its advantages and considerations, and the study aims to evaluate and compare their outcomes comprehensively. Including minimally invasive and robot-assisted surgeries acknowledges the evolving landscape of surgical techniques, aligning with the contemporary emphasis on reducing patient morbidity and improving recovery.

Patients meeting the inclusion criteria will be assessed for eligibility, and the choice of intervention will be based on factors such as tumor characteristics, patient condition, and the surgeon’s expertise.

Qualified and experienced health care professionals will perform the surgical procedures at participating institutions. Data related to the interventions, perioperative details, and postoperative outcomes will be systematically collected through the web-based registry developed for the study.

### Criteria for Discontinuing or Modifying Allocated Interventions

The criteria for discontinuing or modifying allocated interventions in LUCENT are established to ensure participants’ safety and well-being while maintaining the study’s integrity. These criteria are designed to address unforeseen circumstances, emerging patient-related factors, or changes in clinical status. The discontinuation or modification of interventions will be considered under the following events:

- Medical emergencies: If a participant experiences a medical emergency during or after the intervention, the allocated intervention may be discontinued or modified to address the emergent health situation. The treating health care professionals will decide on the situation and prioritize the participant’s health and safety.
- Adverse events (AEs): In the case of unexpected AEs related to the allocated intervention, the study protocol allows for the discontinuation or modification of the intervention to mitigate potential harm to the participant. AEs will be monitored, documented, and reported according to the study’s safety monitoring procedures.
- Participant withdrawal: If a participant chooses to withdraw from the study or requests a change in the allocated intervention, their decision will be respected. Withdrawal may be due to personal reasons, differences in health status, or other factors influencing the participant’s willingness to continue with the allocated intervention.
- Protocol deviation: Any deviation from the study protocol deemed necessary for the participant’s well-being may lead to modification or discontinuation of the allocated intervention. Protocol deviations will be documented and reported according to the study’s data management and reporting procedures.
- Clinical judgment: Health care professionals, in consultation with the principal investigator, may exercise clinical judgment to modify or discontinue the allocated intervention if unforeseen clinical circumstances arise that warrant such actions. Decisions will be made based on the best interests of the participant and in adherence to ethical and safety considerations.
- Loss to follow-up: If a participant loses access to follow-up or cannot adhere to the scheduled assessments and interventions, modifications may be considered based on the available information. It is crucial to note that any discontinuation or change of allocated interventions will be thoroughly documented, and the reasons for such actions will be reported in the study records. Additionally, these decisions will be communicated to relevant stakeholders, including the study participants and institutional review boards, ensuring transparency and adherence to ethical standards. The criteria outlined aim to balance participant safety with the scientific rigor of the study.

### Strategies to Improve Adherence to Interventions

The following strategies will be implemented to optimize and enhance adherence throughout the study:

- Patient education and informed consent: Clear and comprehensive education about the study, its purpose, and the importance of adherence will be provided during the informed consent process.
- Multidisciplinary team engagement: A multidisciplinary health care team, including surgeons, nurses, and support staff, will be involved in patient education and engagement.
- Regular communication: Normal communication channels will be established between health care providers and participants to address any concerns, provide ongoing support, and reinforce the importance of adherence.
- Patient-centered decision-making: Patients will be involved in decision-making regarding their treatment to foster a sense of autonomy and ownership in the intervention process.
- Monitoring and feedback: A robust monitoring system will be implemented to track participant progress and intervention adherence.
- Patient support programs: Support programs, including psychological support and resources, will be offered to address the emotional and psychological aspects of the intervention.
- Use of technology: Technology, such as mobile apps or online platforms, will be leveraged to provide educational materials, reminders, and real-time support.
- Continuous participant engagement: Strategies will be implemented to maintain participant engagement throughout the study, including newsletters, educational materials, and updates on study progress.

### Relevant Concomitant Care Permitted or Prohibited During the Trial

Adherence to interventions is crucial for scientific validity and participant well-being. The following strategies will be implemented to optimize adherence throughout the study: clear and comprehensive education during informed consent, and emphasizing the study’s purpose and the importance of adherence. Patients will be informed about the potential benefits, highlighting their contribution to advancing knowledge in lung cancer treatment. A multidisciplinary health care team, including surgeons, nurses, and support staff, will be involved in patient education and engagement. A collaborative approach ensures consistent information and support from various health care professionals. Treatment plans will be tailored to individual patient needs and preferences to enhance buy-in and motivation to adhere to the protocol. Patient-specific factors, such as lifestyle and cultural background, can improve adherence. Regular communication channels will be established between health care providers and participants to address concerns, provide ongoing support, and reinforce the importance of adherence. Telehealth or virtual platforms will be implemented for follow-up consultations to facilitate continuous communication. Patients will be included in decision-making to foster a sense of autonomy and ownership in the intervention process. Shared decision-making can enhance commitment and adherence to the chosen intervention. A robust monitoring system will be implemented to track participant progress and adherence. Timely feedback will be provided to participants on their progress. Acknowledgment of their contributions is considered positive reinforcement. Support programs, including psychological support and resources, will be offered to address the emotional and psychological aspects of the intervention. Support programs help patients to cope with challenges, fostering a positive attitude toward the intervention. Technologies, such as mobile apps and online platforms, will be adopted to provide educational materials, reminders, and real-time support. Technology can enhance engagement and facilitate communication between participants and health care providers. Flexibility will be offered in scheduling interventions to accommodate individual participant preferences and logistical considerations. Flexible scheduling can reduce barriers to adherence and increase overall participant satisfaction. Strategies will be implemented to maintain participant engagement, including newsletters, educational materials, and updates on study progress. This will ensure that participants feel connected and valued as contributors to the research endeavor.

### Provisions for Posttrial Care

The provisions for posttrial care in LUCENT are designed to ensure that participants receive appropriate medical attention and support even after their active involvement in the study has concluded. The posttrial care plan prioritizes the well-being of participants and addresses any ongoing medical needs that may arise. The key provisions for posttrial care include the following:

- Continuity of standard medical care: Participants will continue to receive standard medical care for lung cancer as per established clinical guidelines, irrespective of their involvement in the study. The study team will facilitate the seamless transition of participants back to routine medical care provided by their health care providers.
- Access to study-related information: Participants will continue to have access to information related to the study, including summaries of study results, as they become available and appropriate for dissemination.
- Follow-up assessments: If any scheduled follow-up assessments or evaluations are part of the study protocol extending beyond the active trial period, participants will be informed about and encouraged to attend these follow-up appointments.
- AE monitoring: During the posttrial period, the study team will continue to monitor and address any AEs or complications related to the study interventions or procedures.
- Referral to specialists: If participants require specialized care or additional medical attention beyond the scope of the study, appropriate referrals to specialists will be facilitated.
- Communication and support: Participants will be provided with the contact information of the study team or a designated point of contact, allowing them to reach out with any questions or concerns about their participation or posttrial care.
- Emergency procedures: Clear procedures will be in place to manage any emergent medical situations that participants may face after the conclusion of the trial, with appropriate guidance on seeking urgent medical attention. The posttrial care provisions emphasize the ethical responsibility of the study team to prioritize participant welfare beyond the active study period. By providing ongoing support, access to information, and coordination with standard medical care, the study aims to ensure a comprehensive and ethical approach to the well-being of participants even after their active involvement in the observational study has ended.

### Ethical Considerations

The study protocol has received approval from ISMETT Palermo (IT) (IRRB/04/23 20/04/2023). All participants will provide informed consent before participating in the study. Participants will be informed that study findings may be published, and their confidentiality will be maintained.

## Results

### Outcomes

A comprehensive set of outcomes will be evaluated to gain insights into the clinical, psychosocial, and economic impacts of lung cancer treatment in Italy. The outcomes are categorized into primary and secondary objectives.

#### Primary Objectives

The primary objectives are as follows:

- Performance assessment: (1) Development of national performance benchmarks based on the analysis of risk-adjusted outcomes and processes of care indicators; and (2) Evaluation of individual centers compared to national benchmarks for specific subgroups of patients.

#### Secondary Objectives

The secondary objectives are as follows:

- Economic impact assessment: Examination of the financial implications of innovative technologies or treatment pathways for lung cancer.
- Assessment of health care resource utilization for different surgical medical devices during a single episode of hospitalization.
- Psychosocial impact assessment: Investigation of the psychosocial impact of innovative technologies or treatment pathways, such as minimally invasive surgery.
- Assessment of PROs to determine the impact of new targeted treatments on quality of life.

#### Additional Outcomes and Considerations

The additional outcomes and considerations are as follows:

- Longitudinal clinical outcomes: (1) Analysis of long-term clinical outcomes related to the chosen surgical interventions; and (2) Evaluation of recurrence rates, overall survival, and disease-free survival among participants.
- PROs: (1) Collection of PROs related to well-being, quality of life, and psychosocial factors; and (2) Utilization of online administration of questionnaires to enhance efficiency and data quality.
- Health care resource utilization: (1) Examination of the utilization of health care resources, including hospital stay, postoperative care, and associated costs; and (2) Identification of factors influencing resource utilization and potential areas for improvement.
- Treatment decision patterns: (1) Analysis of the patterns in treatment decisions, considering factors such as tumor characteristics and patient preferences; and (2) Exploration of the factors influencing the choice of surgical interventions.
- Patient characteristics and demographics: (1) Documentation of patient demographics, including age, gender, and other relevant characteristics; and (2) Analysis of how patient characteristics may influence treatment outcomes and decision-making.
- AEs and complications: Monitoring and documentation of AEs and complications related to the chosen interventions; and (2) Evaluation of the safety profile of different surgical modalities.

### Participant Timeline

Recruitment will be conducted from January 01, 2024, to December 31, 2026. Follow-up will be performed for a minimum of 2 years. The study will be completed in the year 2028. The assessments at baseline and follow-up are presented in Table 1.

**Table 1:** Study assessments.

Assessment	Baseline	Follow-up
Visit month (from inclusion)	Yes	No
Informed consent	Yes	No
Check if patient meets the inclusion/exclusion criteria	Yes	No
Medical history	Yes	No
Physical examination, including weight	Yes	No
Hematology	Yes	No
Blood chemistry	Yes	No
Radiological assessment	Yes	No
Clinical variables	No	Yes
Health-related quality of life	Yes	Yes
Direct and indirect costs	Yes	Yes

### Sample Size

LUCENT adopts a nonspecific sample size approach, allowing for the inclusion of eligible participants based on the defined criteria. The study aims to enroll a diverse and representative sample of lung cancer patients diagnosed in early and locally advanced stages to capture a comprehensive range of outcomes and experiences. Participants meeting the inclusion criteria will be recruited consecutively from participating institutions to ensure a systematic and unbiased selection process. The study will involve multiple centers across Italy to enhance the diversity and generalizability of the study findings. Data collection will be facilitated through a web-based registry developed for the study, allowing for standardized and centralized data collection from various participating sites.

### Recruitment

Recruitment involves the following:

- Collaboration with health care institutions: Forge partnerships with a network of health care institutions across Italy.
- Multidisciplinary team engagement: Involve a multidisciplinary team, including thoracic surgeons, oncologists, and research staff, in the recruitment process.
- Patient advocacy groups: Partner with patient advocacy groups focused on lung cancer. Leverage these groups to disseminate information, support recruitment efforts, and enhance community trust.
- Continuous communication: Maintain open and continuous communication with potential participants throughout the recruitment period.
- Address concerns, provide clarifications, and emphasize the value of their contribution to research.
- Regular site meetings: Conduct regular meetings with site staff to share best practices, address challenges, and reinforce recruitment strategies. Foster a collaborative and motivated research team.

The study aims to achieve adequate participant enrollment by combining these strategies, ensuring a diverse and representative sample to meet the target sample size within the specified recruitment period.

### Assignment of Interventions: Allocation

Sequence generation, concealment mechanism, and implementation are not applicable as this is a nonrandomized observational study.

### Assignment of Interventions: Blinding

The blinding approach is as follows:

- Participants: Owing to the nature of the surgical intervention, participants and those directly involved in their care can access the assigned intervention. It is challenging to blind participants and health care providers involved in surgical procedures.
- Care providers: Surgeons, health care providers, and clinical care teams responsible for delivering the interventions will not be blinded. Their awareness of the assigned surgical approach is essential for providing appropriate care.
- Outcome assessors: Outcome assessors will be blinded to the assigned interventions, particularly those collecting objective clinical data and endpoints. This includes individuals responsible for assessing recurrence rates, overall survival, and disease-free survival.
- Data analysts: Data analysts involved in the statistical analysis of study outcomes will be blinded to the assigned interventions. Blinding at this stage helps ensure unbiased data interpretation.
- Statistical team: The statistical team responsible for analyzing and interpreting the study results will conduct blinded analyses to prevent potential bias in reporting.
- Data monitors and auditors: To maintain objectivity, data and external auditors may also be blinded to treatment assignments during monitoring and auditing activities.

### Procedure for Unblinding if Needed

Unblinding will only occur under predefined circumstances where knowledge of the treatment assignment is deemed crucial for participant safety or well-being. Only authorized personnel, such as a designated unblinding officer, will access confidential information regarding treatment assignment. The unblinding officer will be independent and not involved in the study’s day-to-day management or the participants’ direct care. The unblinding process will be secure, ensuring only authorized personnel can access information that reveals treatment assignment. Access to unblinded information will be password-protected and restricted to authorized individuals. The unblinding officer will maintain updated emergency contact information for each participant. In an emergency, the unblinding officer can quickly and efficiently communicate the relevant treatment assignment to the appropriate health care providers. All instances of unblinding, including the reason for unblinding, will be thoroughly documented. Documentation will include the date, time, individuals involved, and the specific circumstances necessitating unblinding. A clear communication plan will be established to ensure that unblinding occurs promptly and reaches the relevant individuals in emergencies. Records of unblinding events will be securely stored and kept separate from the primary study database. Access to these records will be restricted to authorized personnel and regulatory authorities, as necessary. Unblinding events and the reasons for unblinding will be promptly reported to the relevant ethical review boards and regulatory authorities. Following any unblinding event, the study team will reassess the blinding procedures to determine if modifications are needed to prevent similar situations in the future. Participants involved in an unblinding event will be promptly notified of the situation, and clear and accurate information about the intervention they received will be provided.

### Data Collection and Management

The plans for the assessment and collection of outcomes are as follows:

- Longitudinal clinical outcomes: Regarding assessment, long-term clinical outcomes, including recurrence rates, overall survival, and disease-free survival, will be assessed through regular follow-up visits and medical records review. Regarding collection, outcome assessors will collect data during scheduled follow-up visits, and data entry personnel will ensure accurate and timely recording in the study database.
- PROs: Regarding assessment, patient-reported well-being and quality of life outcomes will be assessed using online questionnaires. Regarding collection, participants will be provided access to secure online questionnaire-completion platforms. Data will be automatically recorded in the study database.
- Health care resource utilization: Regarding assessment, health care resource utilization, including hospital stay and postoperative care, will be assessed through medical records and billing data. Regarding collection, data collection personnel will extract relevant information from medical records, and billing data will be obtained and recorded for economic analyses.
- Treatment decision patterns: Regarding assessment, patterns in treatment decisions, considering tumor characteristics and patient preferences, will be assessed through medical records and clinician interviews. Regarding collection, data collection personnel will document treatment decisions in the study database based on comprehensive reviews of medical records.
- Economic and psychosocial impact: Regarding assessment, economic and psychosocial impact assessments will be conducted through standardized tools and interviews. Regarding collection, trained personnel will administer economic and psychosocial assessments, and the results will be recorded in the study database.
- AEs and complications: Regarding assessment, continuous monitoring and medical records review will assess AEs and complications. Regarding collection, data collection personnel and safety monitoring teams will document AEs and complications in the study database.
- Stratification factors: Regarding assessment, stratification factors, including tumor stage and patient demographics, will be assessed at baseline. Regarding collection, data collection personnel will collect baseline information during participant enrollment and document it in the study database.
- Data quality assurance: Regarding assessment, regular audits and validation checks will continuously monitor data quality. Regarding collection, data management personnel will conduct routine quality checks to ensure the accuracy and completeness of the collected data.
- Data security and confidentiality: Regarding assessment, data security and confidentiality measures will be assessed through regular reviews and audits. Regarding collection, data management personnel will implement encryption, access controls, and other security measures to protect participant information.
- Reporting and analysis: Regarding assessment, reporting and analysis plans will be periodically reviewed and updated as needed. Regarding collection, the statistical team will conduct blinded data analyses to generate reports for internal and external stakeholders.

The plans to promote participant retention and completion of follow-up are as follows:

- Participant engagement initiatives: Implement a comprehensive plan to maintain ongoing communication and involvement. Regularly update participants on the study progress, key findings, and importance of their continued participation.
- Clear communication of study benefits: Communicate the potential benefits of the study to participants. Emphasize the contribution of their data in advancing scientific knowledge and improving lung cancer treatment strategies.
- Educational materials: Develop and distribute educational materials explaining the importance of follow-up assessments. Provide resources that enhance participants’ understanding of the study’s objectives and their role in contributing to research.
- Participant reminders: Implement a system of reminders for upcoming follow-up visits, assessments, and questionnaire completions. Use multiple communication channels, including emails, phone calls, and text messages, to ensure participants are aware of and prepared for scheduled activities.
- Flexible follow-up options: Offer flexible options for follow-up assessments, including virtual visits or alternative locations. Accommodate participant preferences to reduce barriers to attendance.
- Incentives for follow-up: Provide reasonable incentives for participants to complete follow-up assessments. Ensure that incentives comply with ethical guidelines and do not compromise the voluntary nature of participation.
- Dedicated study coordinator: Study coordinators will be a consistent point of contact, addressing participant queries and concerns.
- Continuous feedback loop: Establish a constant loop by soliciting participant feedback on their experiences.

### Data Management

The LUCENT data management process adheres to rigorous standards to ensure accuracy, integrity, and confidentiality. Standardized data collection forms will gather information consistently across all participating centers. Electronic data capture systems will be employed for efficient and accurate data entry. Regular checks and validations will be implemented to ensure the accuracy and completeness of the collected data. Data quality will be systematically monitored to identify and promptly address any discrepancies or errors. Secure and encrypted servers will be used to store electronic data. Access controls will be implemented to restrict unauthorized access to sensitive information. Stringent security measures, including encryption and secure authentication, will safeguard participant data. Compliance with relevant data protection regulations and guidelines will be maintained. Data will be shared according to appropriate ethical and legal frameworks if applicable. Access to data will be regulated to ensure privacy and confidentiality. A data monitoring committee will ensure that the study is conducted with the highest ethical and scientific standards.

### Confidentiality

The aspects of confidentiality are as follows:

- Participant confidentiality: Assign unique identifiers to participants to anonymize data while maintaining linkage to individual records internally. Implement strict access controls to restrict data access to authorized personnel only. Define user roles and permissions based on the principle of least privilege to limit access to specific data sets.
- Data encryption: Apply encryption protocols to protect stored and transmitted data. Encrypt sensitive information to prevent unauthorized access in case of a security breach.
- Confidentiality agreements: All personnel involved in the study must sign confidentiality agreements. Emphasize the importance of respecting participant privacy and the confidential nature of study data. Store physical and electronic data in secure environments. Implement measures such as locked cabinets for physical documents and secure servers for electronic data.

### Plans for Collection, Laboratory Evaluation, and Storage of Biological Specimens for Genetic or Molecular Analysis in This Trial/Future Use

There will be no collection, laboratory evaluation, or storage of biological specimens.

### Statistical Methods

#### Statistical Methods for Primary and Secondary Outcomes

The demographic and baseline characteristics and follow-up outcomes will be described as mean, SD, median, and range for continuous variables, and percentage for categorical variables. Descriptive statistics will be calculated for the whole sample and by relevant subgroups (age, gender, diagnosis, etc). Statistically significant differences across groups will be detected using 1-way ANOVA. Multiple linear regression will be performed to investigate the impact of participant characteristics on short- and medium-term outcomes.

This study will use the official Italian EQ-5D-5L questionnaire to measure health and quality of life. The EQ-5D-5L descriptive system includes 5 dimensions: mobility (MO), self-care (SC), usual activities (UA), pain/discomfort (PD), and anxiety/depression (AD). Each dimension is articulated into 5 severity levels: no problems, slight problems, moderate problems, severe problems, and extreme problems (or unable to). Consequently, 3125 (55) possible health states are determined by response combinations and identified with a unique 5-digit number ranging from perfect health (“11111”) to the worst state (“55555”). Each health state will be converted into a single index value from 0 (assigned to death) to 1 (perfect health state), using predefined preference weights collected at the population level.

All statistical analyses will be performed using R software (R Project for Statistical Computing).

#### Interim Analyses

Interim analyses will be conducted at predetermined intervals to assess the study’s safety, efficacy, and futility. The analyses will be planned to coincide with critical milestones, such as the completion of a specific percentage of follow-up assessments or the occurrence of a predefined number of events. An independent data monitoring committee will be established to oversee interim analyses. The data monitoring committee will consist of experts in the field, statisticians, and clinicians who are not directly involved in the day-to-day conduct of the study. The data monitoring committee will have exclusive access to interim results. Unblinded provisional data will be shared with the data monitoring committee for their assessment of safety, efficacy, and overall study progress.

Clear stopping guidelines will be established in advance, outlining the criteria for stopping the trial. Controlling guidelines will include considerations for both safety and efficacy, with predefined thresholds for statistical significance. The trial may be stopped for the following reasons:

- Efficacy: If interim analyses demonstrate overwhelming evidence of benefit, meeting predetermined criteria for early success.
- Safety: If there is a significant safety concern, meeting predefined criteria for harm.
- Futility: If interim analyses indicate that the study is unlikely to achieve its primary objectives.

The principal investigator and the steering committee will take the final decision to terminate the trial. The steering committee, comprising key study investigators, will consider the data monitoring committee’s recommendations and ultimately take a decision.

A clear communication plan will be in place to ensure timely and transparent reporting of interim results to relevant stakeholders. Communication channels will be predefined to facilitate the efficient dissemination of information. Any decision to stop the trial will be promptly reported to relevant ethical review boards and regulatory authorities. Transparent reporting ensures compliance with ethical standards and regulatory requirements.

#### Methods for Additional Analyses

Other studies, including subgroup and adjusted analyses, will explore variations in treatment effects among different patient subgroups. Adjusted analyses will account for potential confounding factors, such as baseline characteristics and comorbidities, that may influence study outcomes. Statistical methods, such as regression models and stratification, will be employed to assess the impact of variables on study outcomes.

Predefined subgroup analyses will be based on key demographic and clinical variables, such as age, tumor stage, and treatment modality. Criteria for conducting subgroup analyses will be established to avoid data-driven post hoc analyses, and exploratory analyses may be performed to generate hypotheses for future research. Sensitivity analyses will assess the robustness of findings by varying assumptions or statistical methods.

Multiplicity issues in subgroup analyses will be addressed by adjusting significance levels and applying appropriate statistical corrections, such as Bonferroni adjustments, to control for type I errors. Guidelines for interpreting additional analyses will be established, distinguishing between confirmatory and exploratory findings. Reporting standards will be adhered to, and results will be presented with effect sizes, CIs, and *P*-values for transparency and reproducibility.

The findings of additional analyses will be reviewed to ensure methodological rigor and validity. Publication policies will be defined, and any deviations from the original study protocol will be disclosed. Additional analysis findings will be communicated to relevant stakeholders, including study sponsors, regulatory authorities, and the scientific community, providing context for interpretation and discussing the implications on the overall study results.

#### Methods in Analysis to Handle Protocol Nonadherence and Any Statistical Methods to Handle Missing Data

The primary analysis will adhere to the intention-to-treat (ITT) principle with consideration of participants based on their randomized treatment assignment. Protocol nonadherence, such as deviations from the assigned treatment, will be captured and addressed in sensitivity analyses, including per-protocol analyses.

Sensitivity analyses will be conducted to assess the impact of protocol nonadherence on study outcomes. These analyses may include per-protocol analyses, where only participants strictly adhering to the protocol are considered, providing insights into the robustness of the primary ITT analysis.

Multiple imputation methods will be employed to handle missing data. Missing data patterns will be carefully examined, and imputation models will be used to estimate missing values based on observed data, enhancing the completeness of the data set. The missing data mechanism will be assessed to inform the selection of appropriate imputation methods with consideration of mechanisms such as missing completely at random (MCAR), missing at random (MAR), or missing not at random (MNAR).

Imputation models that account for variables associated with missing data, including baseline characteristics, treatment assignment, and relevant covariates, will be developed. The number of imputations will be specified to ensure sufficient imputations to capture uncertainty associated with missing data, commonly using 5 or 10 imputations.

Sensitivity analyses will compare results from the imputed data set with those from observed data to assess the impact of imputation on study outcomes. The methods used to handle missing data will be reported in study publications, transparently communicating any assumptions made during imputation and their potential implications on the study findings.

External validation of imputation models will be considered to enhance the generalizability of imputed data. Collaboration with statistical experts will be sought to ensure the appropriateness and rigor of the chosen methods for handling missing data, with input from experts during the planning phase to adapt methods as needed based on recommendations.

#### Plans to Give Access to the Full Protocol, Participant-Level Data, and Statistical Code

LUCENT has plans to make the full study protocol publicly accessible, allowing interested parties to review the details of the study. Additionally, the intention is to grant public access to the participant-level data set, promoting transparency and openness in sharing study data. The statistical code used for data analysis will also be made available to the public, allowing researchers and analysts to understand and evaluate the study’s statistical methods.

For dissemination, reputable data-sharing platforms or repositories will be considered to host the protocol, participant-level data set, and statistical code. The study will ensure compliance with ethical standards and legal requirements when granting public access, prioritizing participant confidentiality. Evaluation of embargo periods before public release may allow researchers involved in the study to publish primary findings before more comprehensive access is granted.

Clear documentation outlining the terms and conditions for using the participant-level data set and statistical code will be developed. This includes guidelines to ensure responsible and ethical use of the shared data. To enhance the visibility and accessibility of study-related information, collaboration with reputable data repositories or platforms specializing in hosting and sharing research data will be sought.

Effective communication strategies will be implemented to inform the research community and the public about the availability of the study protocol, participant-level data set, and statistical code. Continuous updates will ensure the most recent protocol version and related materials are accessible to the public. The study aims to foster transparency, collaboration, and reproducibility in research by sharing key study components openly.

### Oversight and Monitoring

The composition of the coordinating center and trial steering committee will be decided. Moreover, the composition of the data monitoring committee, its role, and its reporting structure will be decided.

### AE Reporting and Harm

A systematic approach will be employed to collect AEs, encompassing solicited and spontaneously reported events. A standardized assessment process will be in place to evaluate the severity, causality, and expectedness of each noted AE, with clearly defined criteria for categorizing events as serious AEs.

Reporting mechanisms for solicited and spontaneously reported AEs will be established, and investigators and site personnel will receive training on proper reporting procedures. The study will have a robust system for managing AEs, including immediate actions for severe or unexpected events. It will provide appropriate medical intervention and follow-up for affected participants.

Thorough documentation of each AE will be maintained, including details about onset, duration, resolution, and any actions taken. Data on AEs will be included in study reports and publications. AEs meeting the criteria for reporting to ethics committees and regulatory authorities will be promptly communicated following local regulations and ethical guidelines.

Consideration may be given to establishing a safety monitoring committee to independently review and assess safety-related data and provide recommendations for the trial’s continuation, modification, or termination based on safety considerations.

During the informed consent process, participants will be informed about potential risks and AEs associated with the trial interventions. Regular communication with participants will be maintained to encourage reporting any AEs experienced during the study.

A comprehensive data safety and monitoring plan will be developed, outlining procedures for collecting, assessing, reporting, and managing AEs. The plan will be followed diligently to ensure the highest standards of participant safety.

AE reporting procedures will undergo continuous review, allowing for adaptation based on emerging safety data or changes in the risk-benefit profile of the trial interventions.

### Frequency and Plans for Auditing Trial Conduct

Trial conduct will be audited at planned intervals throughout the study. Procedures for auditing will be detailed to comprehensively examine aspects such as protocol adherence, data integrity, participant safety, and regulatory compliance. The auditing process will be independent of investigators and the sponsor, possibly involving external auditors or separate entities to maintain objectivity.

The scope of audits will cover various aspects, including adherence to the study protocol, informed consent processes, data collection and management procedures, monitoring of AEs, and regulatory compliance. Site selection for audits will be based on risk assessment with consideration of factors like the number of enrolled participants, data quality, and historical site performance, with both random and targeted site selection strategies.

Comprehensive audit reports will be generated, documenting findings, observations, and any identified deviations from the study protocol or regulatory requirements. Recommendations for corrective actions will be included in these reports. In response to audit findings, corrective and preventive measures will be implemented promptly, with collaboration between the study team and auditors to address identified issues.

Audit results, including findings and actions taken, will be communicated to relevant stakeholders to ensure transparency and accountability. The auditing process will be part of the continuous monitoring and improvement framework of the study, informing ongoing quality assurance measures to enhance the overall integrity of the trial. As required, significant audit findings impacting participant safety or data integrity will be reported to regulatory authorities to maintain regulatory compliance.

### Plans for Communicating Important Protocol Amendments to Relevant Parties

A transparent communication strategy will be employed to disseminate information about essential protocol amendments. Significant changes will be notified directly to principal investigators, site personnel, and ethical committees (research ethics committees/institutional review boards). Participants will be informed of modifications that may impact their involvement, emphasizing explicit and understandable communication to maintain trust.

Trial registries will be promptly updated to reflect any protocol changes, ensuring accurate documentation of amendments, including modifications to eligibility criteria, outcomes, and analyses. Journals and publications, if applicable, will be notified of protocol amendments to align with reporting guidelines, and manuscripts will accurately reflect the final protocol.

Regulatory authorities will be informed following local regulations, maintaining timely and compliant reporting for regulatory approval and oversight. Continuous updates will be provided to all relevant parties, ensuring awareness of the study’s evolving nature.

Thorough documentation of protocol amendments and associated communications will be maintained for internal use, audits, and regulatory inspections. A feedback mechanism may allow stakeholders to seek clarification or provide input on protocol changes, fostering open communication and collaboration within the research environment.

### Dissemination Plans

The main results of this clinical study will be published in a peer-reviewed scientific journal.

One of the study chairs will write the final publication based on the final analysis performed by the principal investigators.

Co-authors will be the study’s principal investigators who participate in designing and drawing up the research project. Investigators of foreign centers may be included as authors depending on their contribution to patient assessments and scientific input.

## Discussion

The protocol outlined in LUCENT presents a robust framework for conducting a comprehensive observational study focused on lung cancer patients in Italy. The study’s emphasis on real-world data collection and patient registries provides a valuable opportunity to gather longitudinal data on various aspects of patient care and outcomes, particularly in the context of minimally invasive surgery for lung cancer. One key strength of the protocol is its adherence to the ITT principle, ensuring that participants are analyzed based on their randomized treatment assignment. By incorporating sensitivity analyses to address protocol nonadherence and employing multiple imputation methods for handling missing data, the study demonstrates a commitment to rigor and transparency in data analysis. Furthermore, the protocol’s plan for auditing trial conduct at regular intervals, independent of investigators and sponsors, underscores the importance of maintaining data integrity, participant safety, and regulatory compliance throughout the study. The comprehensive audit reports and corrective actions in response to findings reflect a proactive approach to quality assurance and risk management. The protocol also highlights the importance of transparent communication regarding protocol amendments, ensuring that relevant parties are informed of any significant changes that may impact the study’s conduct or outcomes. By prioritizing clear and explicit communication with participants, ethical committees, and other stakeholders, the study aims to uphold trust and accountability in its research practices. Regarding data sharing and dissemination, the protocol outlines plans to make the full study protocol, participant-level data set, and statistical code publicly accessible. By leveraging reputable data-sharing platforms and repositories, the study aims to promote research transparency, collaboration, and reproducibility while prioritizing participant confidentiality and ethical data use. Overall, the LUCENT protocol demonstrates a comprehensive and meticulous approach to conducting an observational study in lung cancer research. By incorporating robust methods for data analysis, quality assurance measures, and transparent communication strategies, the study sets a strong foundation for generating valuable insights into the outcomes of lung cancer patients undergoing minimally invasive surgery in Italy.

While the LUCENT protocol presents a comprehensive framework for investigating the outcomes of lung cancer patients in Italy, several limitations should be considered. The study’s reliance on voluntary participation may introduce selection bias, as patients who agree to participate will differ systematically from those who decline. This could impact the generalizability of the findings to the broader population of lung cancer patients. Despite rigorous data collection processes, the accuracy and completeness of the collected data may be subject to human error or inconsistencies across participating centers. The study’s focus on lung cancer patients in Italy may limit the generalizability of the findings to other populations with different health care systems, demographics, or treatment practices. Maintaining high follow-up rates over the study duration may be challenging, particularly if patients experience barriers to participation or drop out due to personal reasons. Lower follow-up rates could introduce bias and affect the validity of longitudinal outcome assessments. The absence of external funding for LUCENT may pose challenges regarding resource allocation, data collection, and study implementation. Limited resources could impact the scale and scope of the study, potentially affecting its ability to achieve comprehensive and robust outcomes. While the protocol emphasizes obtaining informed consent and ethical approval, ethical considerations concerning data privacy, participant confidentiality, and potential risks to participants’ well-being must be monitored and addressed throughout the study. The study’s reliance on specific surgical approaches and centers in Italy may limit the external validity of the findings, particularly in regions with different health care infrastructure or surgical practices. Collaboration with international partners could enhance the study’s external validity.

## Appendix

Multimedia Appendix 1 Collaborating institutions.

Multimedia Appendix 2 SPIRIT checklist.

## Abbreviations

AE: adverse event
GCP: Good Clinical Practice
HRQoL: health-related quality of life
ITT: intention-to-treat
LUCENT: Italian Lung Cancer Observational Study
NSCLC: non-small cell lung cancer
PRO: patient-reported outcome

## Data Availability

Data and materials related to the study are available upon request.
