# Correction: Outcomes of Patients With Early and Locally Advanced Lung Cancer: Protocol for the Italian Lung Cancer Observational Study (LUCENT)

**DOI:** 10.2196/67629

**Published:** 2024-10-29

**Authors:** Luca Bertolaccini, Oriana CIani, Marco Lucchi, Francesco Zaraca, Alessandro Bertani, Roberto Crisci, Lorenzo Spaggiari, LUCENT Study Group

**Affiliations:** 1 Department of Thoracic Surgery IEO, European Institute of Oncology IRCCS Milan Italy; 2 Centre for Research on Health and Social Care Management (CERGAS) Bocconi School of Management Bocconi University Milan Italy; 3 Division of Thoracic Surgery University of Pisa Pisa Italy; 4 Division of Thoracic Surgery Regional Hospital Bonze/Bolzano Italy; 5 Unit of Thoracic Surgery and Lung Transplantation Department for the Treatment and Study of Cardiothoracic Diseases and Cardiothoracic Transplantation Istituto Mediterraneo per i Trapianti e Terapie ad Alta Specializzazione (IRCCS-ISMETT) Palermo Italy; 6 Division of Thoracic Surgery University of L'Aquila L'Aquila Italy; 7 Department of Oncology and Hemato-Oncology University of Milan Milan Italy; 8 see Authors' Contributions

In Outcomes of Patients With Early and Locally Advanced Lung Cancer: Protocol for the Italian Lung Cancer Observational Study (LUCENT)” (JMIR Res Protoc 2024;13:e57183) the authors noted one error.

In the authorship, the following excerpt:

Luca Bertolaccini^1^, Oriana Ciani^2*^, Marco Lucchi^3^, Francesco Zaraca^4^, Alessandro Bertani^5^, Roberto Crisci^6^, Lorenzo Spaggiari^1,7^; LUCENT Study Group^8^

^1^Department of Thoracic Surgery, IEO, European Institute of Oncology IRCCS, Milan, Italy.

^2^Centre for Research on Health and Social Care Management (CERGAS), Bocconi School of Management, Bocconi University, Milan, Italy.

^3^Division of Thoracic Surgery, University of Pisa, Pisa, Italy.

^4^Division of Thoracic Surgery, Regional Hospital, Bozen/Bolzano, Italy.

^5^Unit of Thoracic Surgery and Lung Transplantation, Department for the Treatment and Study of Cardiothoracic Diseases and Cardiothoracic Transplantation, Istituto Mediterraneo per i Trapianti e Terapie ad Alta Specializzazione (IRCCS-ISMETT), Palermo, Italy.

^6^Division of Thoracic Surgery, University of L'Aquila, L'Aquila, Italy.

^7^Department of Oncology and Hemato-Oncology, University of Milan, Milan, Italy.

^8^see Authors' Contributions.

^*^these authors contributed equally

has been revised to:

Luca Bertolaccini^1*^, Oriana Ciani^2*^, Marco Lucchi^3^, Francesco Zaraca^4^, Alessandro Bertani^5^, Roberto Crisci^6^, Lorenzo Spaggiari^1,7^; LUCENT Study Group^8^

^1^Department of Thoracic Surgery, IEO, European Institute of Oncology IRCCS, Milan, Italy.

^2^Centre for Research on Health and Social Care Management (CERGAS), Bocconi School of Management, Bocconi University, Milan, Italy.

^3^Division of Thoracic Surgery, University of Pisa, Pisa, Italy.

^4^Division of Thoracic Surgery, Regional Hospital, Bozen/Bolzano, Italy.

^5^Unit of Thoracic Surgery and Lung Transplantation, Department for the Treatment and Study of Cardiothoracic Diseases and Cardiothoracic Transplantation, Istituto Mediterraneo per i Trapianti e Terapie ad Alta Specializzazione (IRCCS-ISMETT), Palermo, Italy.

^6^Division of Thoracic Surgery, University of L'Aquila, L'Aquila, Italy.

^7^Department of Oncology and Hemato-Oncology, University of Milan, Milan, Italy.

^8^see Authors' Contributions.

^*^these authors contributed equally

The correction will appear in the online version of the paper on the JMIR Publications website on October 29, 2024, together with the publication of this correction notice. Because this was made after submission to PubMed, PubMed Central, and other full-text repositories, the corrected article has also been resubmitted to those repositories.

