# Supplementary material for: Outcomes of Patients With Early and Locally Advanced Lung Cancer: Protocol for the Italian Lung Cancer Observational Study (LUCENT)
Source: JMIR Res Protoc. 2024 Oct 8;13:e57183. doi: 10.2196/57183 (PMC11496920; doi:10.2196/57183)
Supplement: Multimedia Appendix 1 [file resprot_v13i1e57183_app1.docx]

**Multimedia Appendix 1.** Collaborating institutions.

| **Participant Centres** | | | | | |
| --- | --- | --- | --- | --- | --- |
| **No.** | **Centres** | **Investigators** | | | **City** |
| 1 | UOC Chirurgia Toracica  Azienda ospedaliero-universitaria (AOU) Sant’Andrea  Università Sapienza Roma | Rendina Erino Angelo | Menna Cecilia | Roma | |
| 2 | UOC Chirurgia Toracica  Policlinico Umberto I, Università Sapienza Roma | Venuta Federico | Vannucci Jacopo | Roma | |
| 3 | UOC Chirurgia Toracica Osp Cisanello – Università di Pisa | Lucchi Marco | Ambrogi Macello | Pisa | |
| 4 | UOC Chirurgia Toracica – Osp Mazzini - Università dell’Aquila | Divisi Duilio | Zaccagna Gino | L’Aquila | |
| 5 | Department of Patho-Physiology and Transplantation, University of Milan | Nosotti Mario | Palleschi Alessandro | Milan | |
| 6 | UOC Chirurgia Toracica e dei Trapianti - ISMETT | Bertani Alessandro | Ferrigno Pia | Palermo | |
| 7 | UOC Chirurgia Toracica - Osp "Santa Maria della Misericordia" - Università di Perugia | Puma Francesco | Ceccarelli Silvia | Perugia | |
| 8 | Department of Vascular and Thoracic Surgery,  Central Hospital | Perkmann Reinhold | Zaraca Francesco | Bolzano | |
| 9 | UOC Chirurgia Toracica - AUSL Romagna – Università di Bologna | Stella Franco | Argnani Desideria | Forlì (FC) | |
| 10 | UOC Chirurgia Toracica – Osp Sant’Anna | Dolci Giampiero | Tamburini Nicola | Ferrara | |
| 11 | UOC Chirurgia Toracica Oncologica IRCCS Candiolo | Droghetti Andrea | Cartia Carla | Candiolo (TO) | |
| 12 | UOC Chirurgia Toracica, Presidio Ospedaliero “Eugenio Morelli”, ASST Valtellina e Alto Lario | Scannagatta Paolo | Naldi Giuseppe | Sondalo (SO) | |
| 13 | UOC Chirurgia Toracica Osp Careggi - Dipartimento di Medicina Sperimentale e Clinica, Università di Firenze | Voltolini Luca | Gonfiotti Alessandro | Firenze | |
| 14 | UOC di Chirurgia Toracica - Ospedali Riuniti di Foggia, , Università di Foggia | Sollitto Francesco | Loizzi Domenico | Foggia | |
| 15 | UOC di Chirurgia Toracica - Osp Brotzu | Cherchi Roberto | Ferrari Paolo Albino | Cagliari | |
| 16 | UOC di Chirurgia Toracica - Osp S. Luigi Gonzaga, Università di Torino | Leo Francesco | Sobrero Simona | Orbassano (TO) | |
| 17 | UOC di Chirurgia Toracica Dipartimento di Scienze Mediche Traslazionali - Università Vanvitelli | Fiorelli Alfonso | Natale Giovanni | Napoli | |
| 18 | UOC Chirurgia Generale ad indirizzo Toracico - ASST Settelaghi - Osp Pubblico - Uninsubria | Imperatori Andrea | Di Natale Davide | Varese | |
| 19 | UOC Chirurgia Toracica – Az. Universitario-Ospedaliera – Università di Parma | Carbognani Paolo | Bocchialini Giovanni | Parma | |
| 20 | UOC Chirurgia Toracica - Osp S. Marco | Terminella Alberto | Cusumanno Giacomo | Catania | |
| 21 | UOC Chirurgia Toracica - Osp Cannizzaro | Nicolosi Maurizio | Nicolosi Giuseppe | Catania | |
| 22 | UOC Chirurgia Toracica - Humanitas | Macrì Paolo | Maria Stefania | Catania | |
| 23 | UOC Chirurgia Toracica - Osp Civico | Librizzi Damiano | Raffaele Francesco | Palermo | |
| 24 | UOC Chirurgia Toracica - Osp Cervello | Agneta Giuseppe | Fatica Federica | Palermo | |
| 25 | UOC Chirurgia Toracica – Humanitas Gavazzeni | Bortolotti Luigi | Italiani Alberto | Bergamo | |
| 26 | UOC Chirurgia Toracica – Osp. Vito Fazzi | Lopez Camillo | Imbriglio Giovanna | Lecce | |
| 27 | UOC Chirurgia Toracica – Osp Sacro Cuore Don Calabria | Gavezzoli Diego | Perroni Gianluca | Verona | |
| 28 | UOC Chirurgia Toracica - Osp S. Croce e Carle | Denegri Andrea | Scarnecchia Elisa | Cuneo | |
| 29 | UOC Chirurgia Toracica – Osp S. Martino | Pariscenti Gianluca | Manitto Mattia | Genova | |
| 30 | UOC Chirurgia Toracica - Osp del Mare | Cagini Lucui | Pourmolkara Domenico | Napoli | |
| 31 | UOC Chirurgia Toracica - S.C.D.U Molinette – Univerisità di Totino | Ruffini Enrico | Lyberis Paris | Turin | |
| 32 | UOC Chirurgia Toracica – Osp. S. Paolo – Università di Milano | Baisi Alessandro | Mazzuzzo Alessandra | Milan | |
| 33 | Osp. Le Scotte Siena. UOC Chirurgia Toracica, Università di Siena | Paladini Piero | Ghisalberti Marco | Siena | |
| 34 | UOC Chirurgia Toracica – Osp Papardo | Casablanca Giuseppe | D’Agostino Federica | Messina | |
| 35 | UOC Chirurgia Toracica – Osp Civile – Università di Modena | Filosso Pierluigi | Binchi Daniele | Baggiovara (M0) | |
| 36 | Osp. SS Annunziata – UOC Chirurgia Goracica ad indirizzo Toracico - Università di Chieti | Mucilli Felice | Pepe Francesco | Chieti | |
| 37 | UOC Chirurgia Toracica - Osp Cattinara | Cortale Maurizio | Lovadina Stefano | Trieste | |
| 38 | UOC Chirurgia Toracica - Osp San Gerardo | Petrella Francesco | Raveglia Federico | Monza | |
| 39 | - UOC Chirurgia Toracica - Osp Maggiore della carità -Università Piemonte Orientale | Rena Ottavio | Rena Ottavio | Novara | |
| 40 | IEO, European Institute of Oncology IRCCS | Lorenzo Spaggiari | Luca Bertolaccini | Milan | |
